# Supplementary material for: Sex and Age-Based Differences in Immune Responses to a Peptide Vaccine for Melanoma in Two Clinical Trials
Source: Vaccines (Basel). 2025 Feb 16;13(2):194. doi: 10.3390/vaccines13020194 (PMC11860781; doi:10.3390/vaccines13020194)
Supplement: Supplementary file 1 [file vaccines-13-00194-s001.zip › vaccines-3361987-supplementary.pdf]

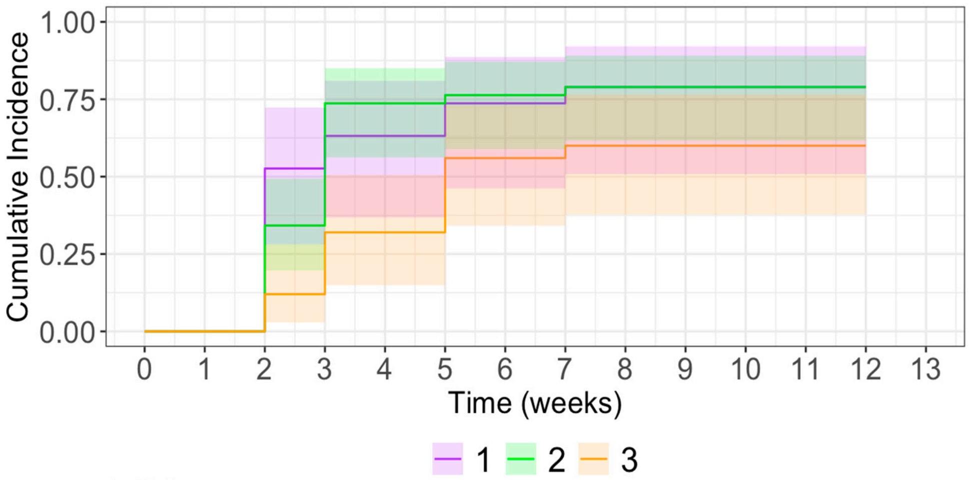

At Risk

1	19	19	19	9	7	7	5	5	2	2	2	2	2	0
2	38	38	38	25	10	10	9	9	6	6	6	6	6	0
3	25	25	25	22	17	17	11	11	4	4	4	4	4	0

Events

1	0	0	10	12	12	14	14	15	15	15	15	15	15	15
2	0	0	13	28	28	29	29	30	30	30	30	30	30	30
3	0	0	3	8	8	14	14	15	15	15	15	15	15	15

Supplemental Figure S1. CumInc of T cell response to 12 melanoma peptides (12MP) after vaccination with 12MP + Tet. Age groupings were less than 46 years (group 1), 46 years to less than 64 years (Group 2), and 64 years and older (Group 3) (p = 0.05).
